# The Buffer Capacity of Polyelectrolyte Microcapsules Depends on the Type of Template

**DOI:** 10.3390/polym16162261

**Published:** 2024-08-09

**Authors:** Alexey V. Dubrovskii, Aleksandr L. Kim, Sergey A. Tikhonenko

**Affiliations:** Institute of Theoretical and Experimental Biophysics Russian Academy of Science, 3, Institutskaya Str., 142290 Puschino, Moscow Region, Russia; dav198@mail.ru (A.V.D.); kimerzent@gmail.com (A.L.K.)

**Keywords:** polyelectrolyte microcapsules, buffer capacity, polystyrene, ionic strength, temperature

## Abstract

One of the key physicochemical parameters of polyelectrolyte microcapsules (PMCs) is their buffer capacity (BC). The BC of the microcapsules allows for an assessment of the change in protonation state across the entire polyelectrolyte system, which directly impacts the buffer barrier of PMCs, as well as the stability and physical properties of their shell. However, the buffer capacity of PMCs and their behavior under changes in ionic strength and temperature can differ depending on the type of core used to form the microcapsules. As part of this study, we revealed the buffer capacity (BC) of polyelectrolyte microcapsules formed on polystyrene cores (PMC_Ps_) and studied the influence of ionic strength and environmental temperature on the BC of these capsules. We found that the buffer capacity of PMC_Ps_ differs from the BC of water at a pH above 8; the addition of sodium chloride leads to an increase in buffer capacity in alkaline conditions, and conversely, thermal treatment leads to its decrease at a pH of 9. The results obtained are different from the BC of polyelectrolyte microcapsules formed on CaCO_3_ cores, which suggests a difference in the physicochemical properties of these types of capsules. The buffer capacity of polyelectrolyte microcapsules depends on the type of template used.

## 1. Introduction

Polyelectrolyte microcapsules (PMCs) are a type of nano-engineered multifunctional structures of spherical shape [1,2], which are obtained by the method of layer-by-layer deposition of oppositely charged polyelectrolytes on spherical particles ranging in size from 60 nm to several micrometers, followed by their dissolution [3,4]. Thanks to the step-by-step formation of microcapsules, their properties and functionality can be tuned according to the desired characteristics for specific tasks [5,6,7,8,9]. As a result, this type of capsule has become widely used in the medical field [10,11,12], the food industry [13,14], the development of smart materials [15,16], and other areas.

One of the key physicochemical parameters of polyelectrolyte microcapsules is their buffer capacity. The buffering capacity of a material is defined as its ability to maintain a constant concentration of H^+^ ions in solution. The study of the buffer capacity of microcapsules permits the evaluation of changes in the protonation state of the entire polyelectrolyte system, which directly impacts the buffer barrier of PMCs, the stability, and physical properties of their shell [17,18,19], as well as the interpretation of results of studies with pH-sensitive encapsulated substances [20,21]. We have previously conducted research on the buffer capacity of polyelectrolyte microcapsules, which were formed on CaCO_3_ cores and consisted of polyelectrolytes polyallylamine and polystyrene sulfonate [22,23,24]. Of the greatest interest among the results of these studies is the increase in the buffer capacity of polyelectrolyte microcapsules with an increase in NaCl concentration and a decrease in buffer capacity with an increase in temperature, as these environmental conditions significantly influence the physicochemical properties of PMCs.

However, the buffer capacity of polyelectrolyte microcapsules and their behavior under changes in ionic strength and temperature can vary depending on the type of core used to form the PMCs. This assumption is based on the fact that the morphology and other properties of the microcapsule shell depend on the template used [25]. In particular, PMCs formed on polystyrene (PS) cores have a pronounced shell, the thickness of which varies from 10 to 60 nm [26], while PMCs formed on a CaCO_3_ core are a polyelectrolyte complex of spherical shape with a complex channel-like structure [27]. Also, their morphology varies differently in response to changes in pH and ionic strength: PMCs on a PS template increase in an alkaline medium and decrease at low pH values [26], while PMCs formed on CaCO_3_ particles do not change their size at different pH values [28,29]; an increase in ionic strength leads to a decrease in the size of PMCs formed on a PS core [30], and microcapsules formed on CaCO_3_ particles do not change in size [31]. In addition, PMCs formed on PS cores have greater resistance to highly alkaline conditions and can withstand pH 12.5 for 30 min [30], while PMCs based on CaCO_3_ degrade instantly [30]. Considering the aforementioned differences in the morphology of PMCs formed on PS and CaCO_3_ cores and their reactions to changes in pH and ionic strength, one cannot extrapolate the behavior of changes in the buffer capacity of PMCs formed on a CaCO_3_ core to the behavior of changes in the buffer capacity of PMCs formed on a PS core, especially if we consider that this type of capsule can be used in areas such as microsensors or drug delivery systems as smart materials [32,33,34], where a change in ionic strength and acidity of the medium is implied. Thus, it is necessary to determine the behavior of the buffer capacity of PMCs formed on a PS core (PMC_Ps_).

In connection with the above, the aim of this study is to determine the presence of buffer capacity in PMCs formed on PS cores and to define the behavior of their BC under varying ionic strength and temperature. The results of this study showed that the buffer capacity of PMC_Ps_ and its change in response to the ionic strength and temperature of the solution are different compared to the BC of polyelectrolyte microcapsules formed on CaCO_3_ cores (PMC_Ca_). Firstly, the buffer capacity of PMC_Ps_ is observed only at a pH above 8, while PMC_Ca_ is observed throughout the studied range. Secondly, the BC of PMC_Ps_ increases with increasing NaCl concentration, while the BC of PMC_Ca_ has a maximum buffer capacity at a sodium chloride concentration of 1 M. Thirdly, increasing the incubation temperature slightly reduces the BC of PMC_Ps_, while the BC of PMC_Ca_ gradually decreases with increasing temperature. The findings indicate that the buffer capacity of polyelectrolyte microcapsules is significantly influenced by the core material employed in their formation.

## 2. Materials and Methods

### 2.1. Materials

Polyelectrolytes sodium polystyrene sulfonate (PSS) and polyallylamine hydrochloride (PAH) with a molecular weight of 70 kDa were purchased from Sigma (St. Louis, MO, USA) (Residual Monomer < 10%). Dimethylformamide (DMFA), sodium chloride, sodium hydroxide, and hydrochloric acid were from “Reakhim”. The percentage content of each chemical is a minimum of 99.9%. Polystyrene particles (5 μm) with COOH groups were purchased from Polymer Latex (Saint Petersburg, Russia).

### 2.2. Preparation of Polyelectrolyte Microcapsules

Polyelectrolyte microcapsules were obtained by layer-by-layer adsorption of negatively or positively charged polyelectrolytes on polystyrene microparticles, followed by their dissolution. Layer-by-layer adsorption of PAH and PSS on the surface of polystyrene microparticles was carried out in polyelectrolyte solutions (concentration 2 mg/mL + 0.5 M NaCl). After each adsorption, polystyrene particles with adsorbed polyelectrolytes were washed three times with 0.5 M NaCl solution, which was necessary to remove unabsorbed polymer molecules. Particles were separated from the supernatant by centrifugation. After applying the necessary number of layers, the polystyrene particles were dissolved in DMFA for 48 h. After removing the polystyrene core, the polyelectrolyte microcapsules were washed three times in a DMFA–water solution. After each of the 3 washes, the water–DMFA ratio in the solution was changed by increasing the amount of water by 10%. The washes and the procedure of increasing the amount of water were repeated until the DMFA was completely removed from the wash solution. The resulting capsules were washed three times with water to remove the DMFA. The size, number, and ζ-potential of the microcapsules were measured using the dynamic light scattering method on a Zetasizer nano ZS device (Malvern, UK).

### 2.3. Temperature Treatment

For heating, the suspension of microcapsules was incubated at the corresponding temperature (60 and 90 °C) in a Lauda Ecoline RE 112 (Lauda, Moscow, Russia) thermostat for 60 min. Following the application of the temperature treatment, the polyelectrolyte microcapsules were incubated at room temperature, with the objective of achieving a suspension temperature equal to that of the ambient environment. The subsequent measurement of the buffer capacity of the polyelectrolyte microcapsules was conducted only after the attainment of room temperature.

### 2.4. Measurement of Buffer Capacity

A suspension of microcapsules (PSS/PAH)_3_ (about 6.6 × 10^9^ microcapsules in 8 mL of water) at room temperature was titrated with solutions of acids or bases in the pH range from 4 to 9. The titration was carried out manually. Acid or base (with a concentration of 0.001 M or 0.005 M) was added to the solution to change the pH of the solution by 0.02 or more. The buffer capacity was calculated using Equation (1) after assessing the slope of the titration curves at each point by the change in pH between the previous and subsequent introductions [35]:

(1) $$BC=\frac{n_{NaOH}\left(i+1\right)-n_{NaOH}\left(i-1\right)}{pH\left(i+1\right)-pH\left(i-1\right)}$$

where $n_{NaOH}$ is the number of moles of NaOH added and $\left(i+1\right)$ and $\left(i-1\right)$ refer to the previous and subsequent injections, respectively.

### 2.5. Statistical Processing

Each sample was measured five times. Due to the peculiarity of titration, it is impossible to achieve the same pH value during titration. Therefore, we combined the buffer capacity values obtained from 5 repeated experiments in steps of 0.1 pH values. The graph shows the mean values of the pooled pH points in 0.1 pH steps (X scale) with the mean buffer capacity value for these points (Y scale), with the standard deviation of both the mean pH value (X error bars) and the mean buffer capacity value (Y error bars).

## 3. Results

The buffer capacity of polyelectrolyte microcapsules allows us to evaluate their ability to maintain a constant hydrogen proton concentration in solution, which is particularly important for maintaining the stability of encapsulated objects such as enzymes [36], fluorescent dyes [20], metal particles [37], etc. We have previously shown the buffer capacity of polyelectrolyte microcapsules formed on a CaCO_3_ core [24]. However, depending on the type of core used, the buffer capacity of the capsule and its response to external chemical stimuli may differ [25].

**Figure 1:**
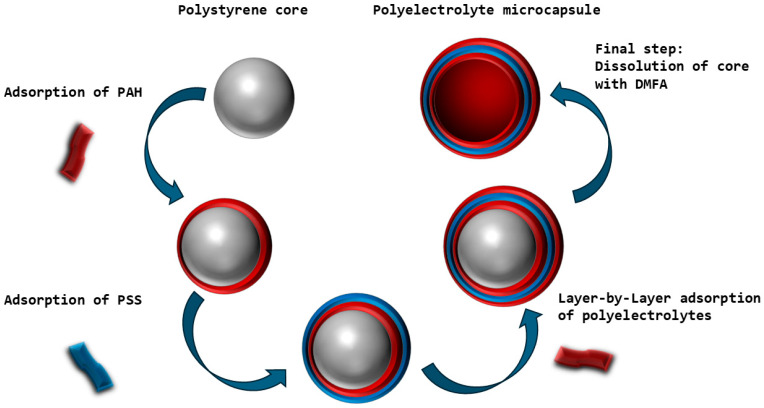
Scheme of preparation of polyelectrolyte microcapsules on a polystyrene core.

In this study, the aim is to determine the presence of buffer capacity (BC) in polyelectrolyte microcapsules formed on a polystyrene (PS) core and to study the behavior of the change in the BC of these capsules under different ionic strengths and temperatures of the solution. For this purpose, polyelectrolyte microcapsules of the composition (PAH/PSS)_3_/PAH were prepared using the method of layer-by-layer adsorption of polystyrene sulfonate (PSS) and polyallylamine (PAH) polyelectrolytes onto a PS particle with covalently bound carboxyl groups. In the final stage of PMC creation, the polystyrene core was removed. The basic scheme for obtaining polyelectrolyte microcapsules is presented in Figure 1.

**Figure 2:**
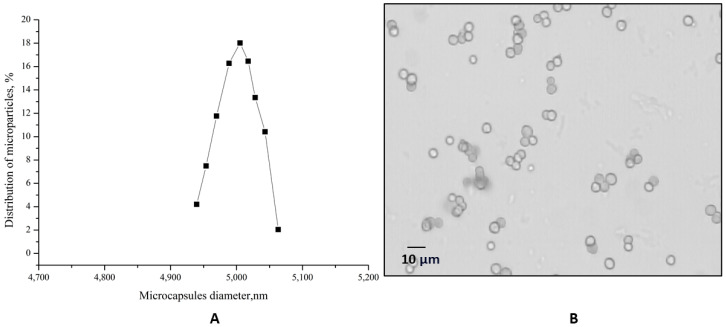
The optical microscopy images of PMCs (**A**). The PMC diameter distribution function (**B**).

The size distribution of the polyelectrolyte microcapsules (PMCs), formed on a polystyrene core (abbreviated as PMC_Ps_), was subsequently determined (Figure 2).

The optical microscopy images of PMCs (Figure 2B) demonstrate the morphological homogeneity of the microcapsules. The microcapsules had an average diameter of 5 μm with a 2.7% polydispersity index (Figure 2A) and an ζ-potential of +26 ± 1 mV.

**Figure 3:**
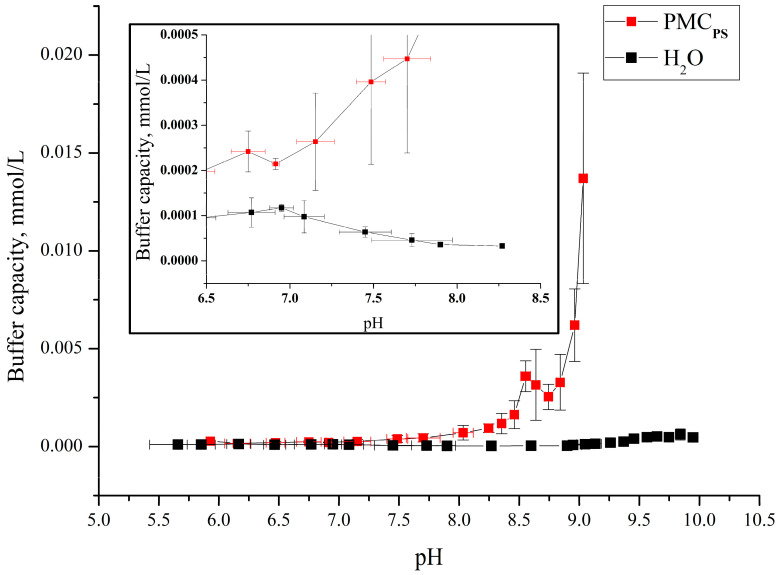
Buffer capacity of PMCs formed on PS with composition (PAH/PSS)_3_/PAH in the pH range from 5.5 to 9.

The obtained polyelectrolyte microcapsules, formed on a polystyrene core, were used to determine their buffer capacity. For this, a suspension of PMCs (6.6 × 10^9^ microcapsules in 8 mL of water), consisting of polyallylamine (PAH) and polystyrene sulfonate (PSS), was titrated with alkali or acid in the pH range from 3.5 to 9, and the change in pH of the suspension was determined using a pH meter. The results of determining the buffer capacity for the pH range from 5.5 to 9 are presented in Figure 3.

**Figure 4:**
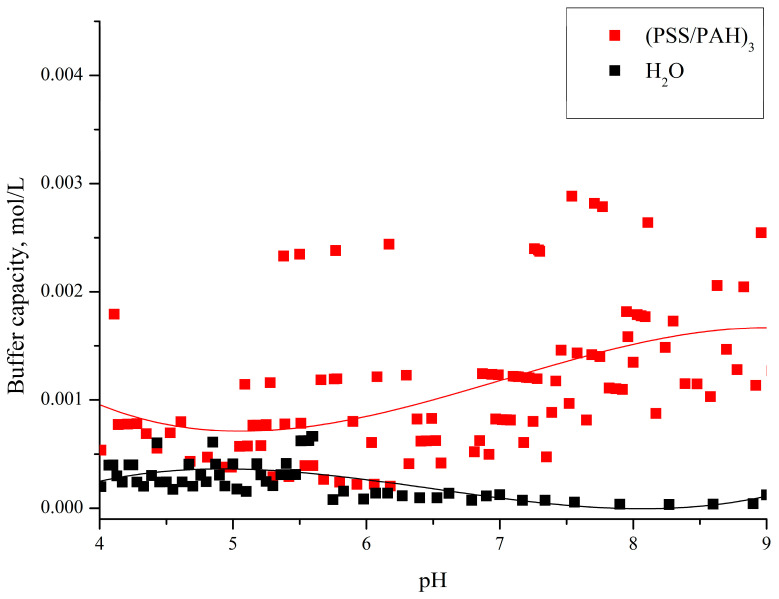
Buffer capacity of microcapsules (PSS/PAH)_3_ and water in the pH range from 4 to 9. Adapted from [24].

From the figure, it can be seen that the buffer capacity of microcapsules formed on PS particles differs from the BC of water at a pH above 8, which is better seen from the insert in Figure 3. In our previous work, we determined the buffer capacity of polyelectrolyte microcapsules formed on a CaCO_3_ core (abbreviated as PMC_Ca_) [24]. Comparing the buffer capacity of these two types of capsules, it was found that the buffer capacity of PMC_Ca_ in the pH range from 5.5 to 9 significantly differs from the presented buffer capacity of PMC_Ps_. The results of our previous work are presented in Figure 4.

From Figure 4, it can be seen that the buffer capacity of PMC_Ca_ monotonically increases with increasing pH from 6, and at pH 9, the BC increases no more than three times. However, for PMC_Ps_, the BC starts to rise only after 8, and at pH 9, the buffer capacity increases 10 times. Considering that the unbound PAH regions are responsible for the buffer capacity of polyelectrolyte microcapsules [23,24], the beginning of the increase in the buffer capacity of PMC_Ca_ at a pH lower than that of PMC_Ps_ may be associated with the fact that PMC_Ps_ have a more stable polyelectrolyte complex of polyallylamine with polystyrene sulfonate than PMC_Ca_. A gradual increase in pH leads to a gradual increase in unbound PAH regions, which leads to an increase in the number of free H^+^ donors and an increase in buffer capacity, which is consistent with our previous research [23,24]. However, PMC_Ps_ show a sharp increase in buffer capacity, which is associated with the simultaneous formation of a larger number of unbound PAH regions than in PMC_Ca_.

**Figure 5:**
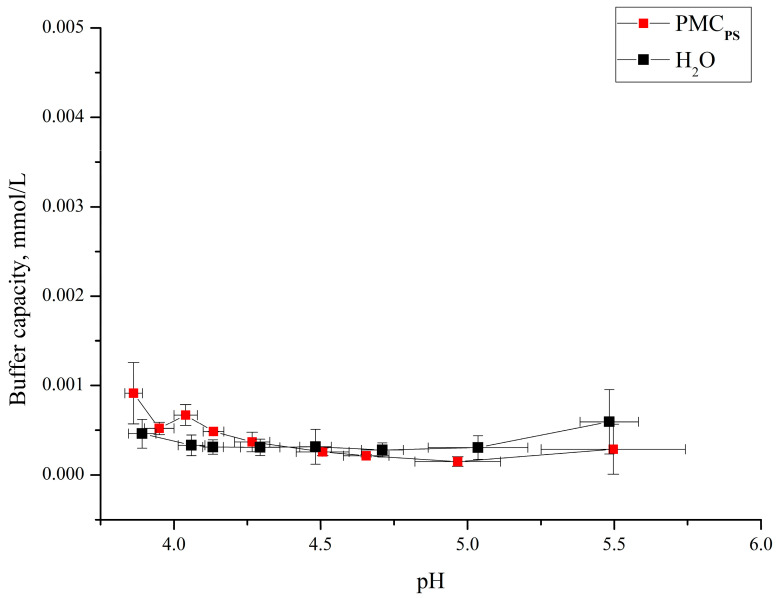
Buffer capacity of PMCs formed on PS with composition (PAH/PSS)_3_/PAH in the pH range from 3.5 to 5.5.

Subsequently, we determined the buffer capacity of PMC_Ps_ in the pH range from 4 to 5.5 and found that in these pH values, PMC_Ps_ do not possess buffer capacity. These results are presented in Figure 5.

As can be seen from Figure 4 and Figure 5, the buffer capacity of PMC_Ps_ and PMC_Ca_ in acidic ranges also differ. This effect can also be explained by the fact that the amount of electrostatically bound polyallylamine with polystyrene sulfonate is significantly larger in PMC_Ps_ than in PMC_Ca_. A larger number of ionic bonds between PSS and PAH chains in PMC_Ps_ is also indirectly confirmed by other works. In particular, several studies have presented the shell thickness for different numbers of layers for PMC_Ps_ and for PMC_Ca_ (without removing cores) [26,27,38]. It was shown that the shell thickness of PMC_Ca_ is 3–5 times larger than in the case of PMC_Ps_, which is explained by the authors by a denser shell (in PMC_Ps_) and, as a result, greater interaction between PSS and PAH.

**Figure 6:**
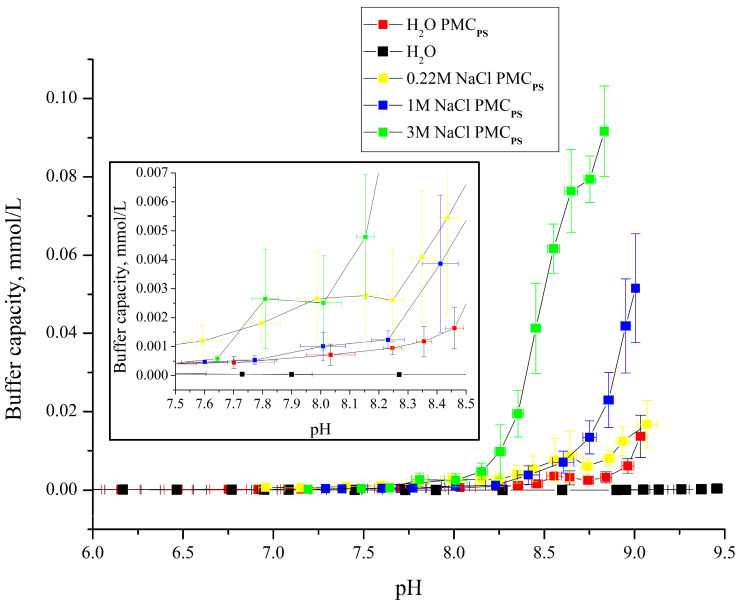
Buffer capacity of PMC_Ps_ composition (PAH/PSS)_3_/PAH at different pH in water, 0.22 M, 1 M, and 3 M NaCl solution.

In the next step, the effect of the ionic strength of the solution on the buffer capacity of polyelectrolyte microcapsules formed on PS cores was studied. This step is necessary to confirm the hypothesis put forward in the previous work [23,24] that the BC of PMCs depends on the number of unbound PAH regions in the composition of the microcapsules since, as the ionic strength of the solution increases, the interaction of polyelectrolytes decreases [39,40,41]. In addition, the obtained results will allow us to take into account the behavior of PMCs and encapsulated substances depending on the level of protonation/deprotonation under different ionic strength conditions. Figure 6 presents the curves of the dependence of the buffer capacity of the PMC_Ps_ composition (PAH/PSS)_3_/PAH on pH in the presence of different concentrations of sodium chloride.

From the figure, it can be seen that the addition of sodium chloride leads to an increase in the buffer capacity of polyelectrolyte microcapsules formed on PS cores, which confirms the hypothesis put forward above. However, the BC of PMC_Ps_ rises with the increase in salt concentration in the range up to 3 M, which does not correspond to the change in the BC of PMC_Ca_ from salt, which we obtained earlier [23,24]. In the previous work, the BC of PMC_Ca_ increased maximally at a salt concentration of 1M, and a further increase in salt concentration did not increase the buffer capacity of this type of capsule. This difference can also be explained by a greater number of PSS-PAH ionic pairs in the PMC_Ps_ shell than in PMC_Ca_, which prevents the breakage of the ionic bond when the ionic strength increases. In addition, this fact is confirmed by the fact that in all described concentrations of NaCl, an increase in the BC of PMC_Ps_ at pH = 7.9 was observed (see insert in Figure 6), and only the BC values differed, which grew with the increase in the ionic strength of the solution.

**Figure 7:**
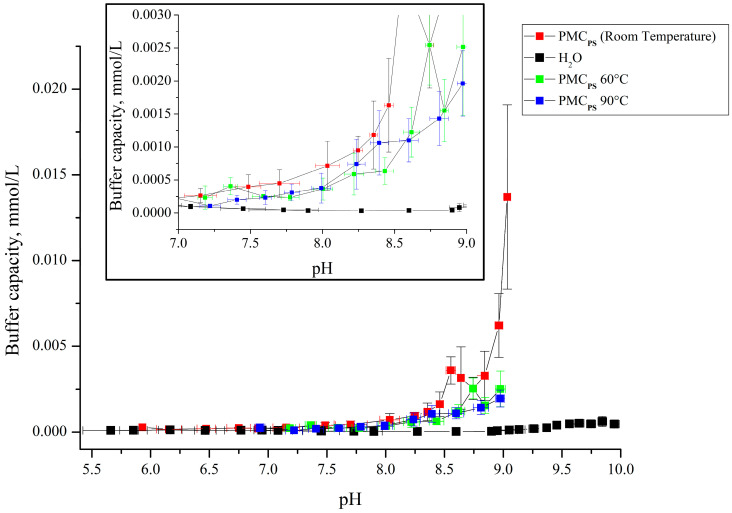
Buffer capacity of PMC_Ps_ composition (PAH/PSS)_3_/PAH at different pH after temperature treatment at 60 and 90 °C.

Subsequently, the effect of temperature exposure on the buffer capacity of polyelectrolyte microcapsules formed on PS cores was studied. Considering the denser arrangement of polyelectrolytes in the PMC_Ps_ shell [26] than in the PMC_Ca_ [27], it can be assumed that the buffer capacity of these two types of capsules will also differ after temperature treatment. In addition, the size of these types of capsules changed differently after their heating: PMC_Ps_ did not change [32,42], and PMC_Ca_ decreased by 24–28% [43]. Figure 7 presents the curves of the dependence of the buffer capacity of the PMC_Ps_ composition (PAH/PSS)_3_/PAH on the pH after temperature treatment at 60 and 90 °C.

From Figure 7, it can be seen that the buffer capacity of thermally treated polyelectrolyte microcapsules formed on a PS core differs only at a pH above 9 from the buffer capacity of non-heated capsules. According to other authors’ works [32,42,43], thermal treatment leads to the breakage and reforming of ionic pairs, which leads to a more compact arrangement of polyelectrolyte molecules. Thus, it is logical to assume that the buffer capacity will change slightly in PMC_Ps_, the polyelectrolytes of which are compactly arranged [26]. However, at pH 9, thermally treated PMC_Ps_ lack a sharp rise in buffer capacity, which suggests an enhancement of the above-described compaction of polyelectrolytes in the shell and an increase in the stability of the polyelectrolyte complex, which allows for reducing the impact of the pH. The previously obtained BC results of PMC_Ca_ [23] after thermal treatment differ from the BC of PMC_Ps_. In the case of PMC_Ca_, the buffer capacity gradually decreased with increasing temperature, while this gradation was absent in PMC_Ps_. This effect can also be explained by the aforementioned compaction of polyelectrolytes.

Based on the above, it can be unequivocally concluded that the buffer capacity of polyelectrolyte microcapsules formed on CaCO_3_ and polystyrene cores differ, and these capsules react differently to changes in ionic strength and temperature of the medium, which depends on the compactness of the polyelectrolyte layers.

## 4. Conclusions

In this study, the buffer capacity (BC) of polyelectrolyte microcapsules formed on polystyrene cores (PMC_Ps_) was determined, and the effect of the ionic strength and medium temperature on the BC of these capsules was studied. It was found that the buffer capacity of PMC_Ps_ differs from the BC of water at a pH above 8; the addition of sodium chloride leads to an increase in buffer capacity in alkaline conditions, and temperature treatment, on the contrary, to its decrease at pH 9. By conducting a comparative analysis with the data we obtained earlier [23,24], differences were identified in the buffer capacity of PMC_Ps_ and its change in response to the ionic strength and temperature of the solution compared to the BC of polyelectrolyte microcapsules formed on CaCO_3_ cores (PMC_Ca_). Firstly, the buffer capacity of PMC_Ps_ is observed only at a pH above 8, while PMC_Ca_ is observed throughout the studied range. Secondly, the BC of PMC_Ps_ increases with increasing NaCl concentration, while the BC of PMC_Ca_ has a maximum buffer capacity at a sodium chloride concentration of 1M. Thirdly, increasing the incubation temperature slightly reduces the BC of PMC_Ps_, while the BC of PMC_Ca_ gradually decreases with increasing temperature. Such differences are presumably related to the more compact arrangement of polyelectrolytes in the PMC_Ps_ shell, which is associated with a greater number of PSS-PAH ionic pairs, complicating the effect of the solution’s pH. It can be concluded that the buffer capacity of polyelectrolyte microcapsules depends on the type of template.

The obtained results excellently demonstrate the difference in the physicochemical properties of polyelectrolyte microcapsules formed on CaCO_3_ and PS cores, which will allow researchers to take into account this difference when choosing a method of encapsulating a substance for the tasks set. In addition, these results will allow consideration of the buffer capacity of PMC_Ps_ when interpreting data when including pH-sensitive compounds in various conditions of ionic strength and temperature.

## Data Availability

Data are contained within the article.

## References

[1] Sukhorukov G.B., Donath E., Davis S., Lichtenfeld H., Caruso F., Popov V.I., Möhwald H. (1998). Stepwise polyelectrolyte assembly on particle surfaces: A novel approach to colloid design. Polym. Adv. Technol..

[2] Sukhorukov G.B., Donath E., Lichtenfeld H., Knippel E., Knippel M., Budde A., Möhwald H. (1998). Layer-by-layer self assembly of polyelectrolytes on colloidal particles. Colloids Surf. A Physicochem. Eng. Asp..

[3] Köhler K., Shchukin D.G., Sukhorukov G.B., Möhwald H. (2004). Drastic Morphological Modification of Polyelectrolyte Microcapsules Induced by High Temperature. Macromolecules.

[4] Gao C., Donath E., Möhwald H., Shen J. (2002). Spontaneous Deposition of Water-Soluble Substances into Microcapsules: Phenomenon, Mechanism, and Application. Angew. Chem. Int. Ed..

[5] Dubreuil F., Elsner N., Fery A. (2003). Elastic properties of polyelectrolyte capsules studied by atomic-force microscopy and RICM. Eur. Phys. J. E.

[6] Sukhorukov G.B., Shchukin D.G., Dong W., Möhwald H., Lulevich V.V., Vinogradova O.I. (2004). Comparative Analysis of Hollow and Filled Polyelectrolyte Microcapsules Templated on Melamine Formaldehyde and Carbonate Cores. Macromol. Chem. Phys..

[7] Köhler K., Shchukin D.G., Möhwald H., Sukhorukov G.B. (2005). Thermal Behavior of Polyelectrolyte Multilayer Microcapsules. 1. The Effect of Odd and Even Layer Number. J. Phys. Chem. B.

[8] Skirtach A.G., Yashchenok A.M., Möhwald H. (2011). Encapsulation, release and applications of LbL polyelectrolyte multilayer capsules. Chem. Commun..

[9] De Geest B.G., Skirtach A.G., Mamedov A.A., Antipov A.A., Kotov N.A., De Smedt S.C., Sukhorukov G.B. (2007). Ultrasound-Triggered Release from Multilayered Capsules. Small.

[10] Verkhovskii R., Ermakov A., Sindeeva O., Prikhozhdenko E., Kozlova A., Grishin O., Makarkin M., Gorin D., Bratashov D. (2021). Effect of Size on Magnetic Polyelectrolyte Microcapsules Behavior: Biodistribution, Circulation Time, Interactions with Blood Cells and Immune System. Pharmaceutics.

[11] Palamarchuk K.V., Borodina T.N., Kostenko A.V., Chesnokov Y.M., Kamyshinsky R.A., Palamarchuk N.P., Yudina E.B., Nikolskaya E.D., Yabbarov N.G., Mollaeva M.R. (2022). Development of Submicrocapsules Based on Co-Assembled Like-Charged Silica Nanoparticles and Detonation Nanodiamonds and Polyelectrolyte Layers. Pharmaceutics.

[12] Gileva A., Trushina D., Yagolovich A., Gasparian M., Kurbanova L., Smirnov I., Burov S., Markvicheva E. (2023). Doxorubicin-Loaded Polyelectrolyte Multilayer Capsules Modified with Antitumor DR5-Specific TRAIL Variant for Targeted Drug Delivery to Tumor Cells. Nanomaterials.

[13] Pan H.M., Subramanian A., Ochs C.J., Dewavrin J.-Y., Beyer S., Trau D.W. (2014). Edible polyelectrolyte microcapsules with water-soluble cargo assembled in organic phase. RSC Adv..

[14] Tan C., Selig M.J., Lee M.C., Abbaspourrad A. (2018). Encapsulation of copigmented anthocyanins within polysaccharide microcapsules built upon removable CaCO_3_ templates. Food Hydrocoll..

[15] Khan A., Sliem M.H., Arif A., Salih M.A., Shakoor R.A., Montemor M.F., Kahraman R., Mansour S., Abdullah A.M., Hasan A. (2019). Designing and performance evaluation of polyelectrolyte multilayered composite smart coatings. Prog. Org. Coat..

[16] Popov A.L., Popova N., Gould D.J., Shcherbakov A.B., Sukhorukov G.B., Ivanov V.K. (2018). Ceria Nanoparticles-Decorated Microcapsules as a Smart Drug Delivery/Protective System: Protection of Encapsulated P. pyralis Luciferase. ACS Appl. Mater. Interfaces.

[17] Musin E.V., Kim A.L., Tikhonenko S.A. (2020). Destruction of polyelectrolyte microcapsules formed on CaCO_3_ microparticles and the release of a protein included by the adsorption method. Polymers.

[18] Lulevich V.V., Vinogradova O.I. (2004). Effect of pH and Salt on the Stiffness of Polyelectrolyte Multilayer Microcapsules. Langmuir.

[19] Kim B., Vinogradova O.I. (2004). pH-Controlled Swelling of Polyelectrolyte Multilayer Microcapsules. J. Phys. Chem. B.

[20] Kazakova L.I., Shabarchina L.I., Sukhorukov G.B. (2011). Co-encapsulation of enzyme and sensitive dye as a tool for fabrication of microcapsule based sensor for urea measuring. Phys. Chem. Chem. Phys..

[21] Kazakova L.I., Shabarchina L.I., Anastasova S., Pavlov A.M., Vadgama P., Skirtach A.G., Sukhorukov G.B. (2013). Chemosensors and biosensors based on polyelectrolyte microcapsules containing fluorescent dyes and enzymes. Anal. Bioanal. Chem..

[22] Musin E.V., Dubrovskii A.V., Kim A.L., Tikhonenko S.A. (2022). A Study of the Buffer Capacity of Polyelectrolyte Microcapsules Depending on Their Concentration and the Number of Layers of the Polyelectrolyte Shell. Int. J. Mol. Sci..

[23] Dubrovskii A.V., Kim A.L., Musin E.V., Tikhonenko S.A. (2022). A Study of the Buffer Capacity of Polyelectrolyte Microcapsules Depending on Their Ionic Environment and Incubation Temperature. Int. J. Mol. Sci..

[24] Dubrovskii A.V., Kim A.L., Musin E.V., Ramazanov B.R., Tikhonenko S.A. (2021). The Discovery of the Buffer Capacity of Various Types of Polyelectrolyte Microcapsules. Polymers.

[25] Kim A.L., Musin E.V., Chebykin Y.S., Tikhonenko S.A. (2024). Characterization of Polyallylamine/Polystyrene Sulfonate Polyelectrolyte Microcapsules Formed on Solid Cores: Morphology. Polymers.

[26] Déjugnat C., Sukhorukov G.B. (2004). pH-Responsive Properties of Hollow Polyelectrolyte Microcapsules Templated on Various Cores. Langmuir.

[27] Volodkin D.V., Petrov A.I., Prevot M., Sukhorukov G.B. (2004). Matrix Polyelectrolyte Microcapsules: New System for Macromolecule Encapsulation. Langmuir.

[28] Tong W., Dong W., Gao C., Möhwald H. (2005). Charge-Controlled Permeability of Polyelectrolyte Microcapsules. J. Phys. Chem. B.

[29] Haložan D., Riebentanz U., Brumen M., Donath E. (2009). Polyelectrolyte microcapsules and coated CaCO_3_ particles as fluorescence activated sensors in flowmetry. Colloids Surf. A Physicochem. Eng. Asp..

[30] Heuvingh J., Zappa M., Fery A. (2005). Salt Softening of Polyelectrolyte Multilayer Capsules. Langmuir.

[31] Pechenkin M.A., Möhwald H., Volodkin D.V. (2012). pH- and salt-mediated response of layer-by-layer assembled PSS/PAH microcapsules: Fusion and polymer exchange. Soft Matter.

[32] Prevot M., Déjugnat C., Möhwald H., Sukhorukov G.B. (2006). Behavior of Temperature-Sensitive PNIPAM Confined in Polyelectrolyte Capsules. ChemPhysChem.

[33] Haložan D., Déjugnat C., Brumen M., Sukhorukov G.B. (2005). Entrapment of a Weak Polyanion and H^+^/Na^+^ Exchange in Confined Polyelectrolyte Microcapsules. J. Chem. Inf. Model..

[34] Radtchenko I.L., Sukhorukov G.B., Leporatti S., Khomutov G.B., Donath E., Möhwald H. (2000). Assembly of Alternated Multivalent Ion/Polyelectrolyte Layers on Colloidal Particles. Stability of the Multilayers and Encapsulation of Macromolecules into Polyelectrolyte Capsules. J. Colloid Interface Sci..

[35] Richard I., Thibault M., De Crescenzo G., Buschmann M.D., Lavertu M. (2013). Ionization Behavior of Chitosan and Chitosan–DNA Polyplexes Indicate That Chitosan Has a Similar Capability to Induce a Proton-Sponge Effect as PEI. Biomacromolecules.

[36] Kim A.L., Musin E.V., Dubrovskii A.V., Tikhonenko S.A. (2019). Determination of urea concentration using urease-containing polyelectrolyte microcapsules. Anal. Methods.

[37] Pavlov A.M., Saez V., Cobley A., Graves J., Sukhorukov G.B., Mason T.J. (2011). Controlled protein release from microcapsules with composite shells using high frequency ultrasound—Potential for in vivo medical use. Soft Matter.

[38] Sukhorukov G.B., Volodkin D.V., Günther A.M., Petrov A.I., Shenoy D.B., Möhwald H. (2004). Porous calcium carbonate microparticles as templates for encapsulation of bioactive compounds. J. Mater. Chem..

[39] Scheepers D., Chatillon B., Borneman Z., Nijmeijer K. (2021). Influence of charge density and ionic strength on diallyldimethylammonium chloride (DADMAC)-based polyelectrolyte multilayer membrane formation. J. Memb. Sci..

[40] Ferrand-Drake del Castillo G., Hailes R.L.N., Dahlin A. (2020). Large Changes in Protonation of Weak Polyelectrolyte Brushes with Salt Concentration—Implications for Protein Immobilization. J. Phys. Chem. Lett..

[41] Sagou J.-P.S., Ahualli S., Thomas F. (2015). Influence of ionic strength and polyelectrolyte concentration on the electrical conductivity of suspensions of soft colloidal polysaccharides. J. Colloid Interface Sci..

[42] Park M.-K., Deng S., Advincula R.C. (2005). Sustained Release Control via Photo-Cross-Linking of Polyelectrolyte Layer-by-Layer Hollow Capsules. Langmuir.

[43] Dubrovskii A.V., Shabarchina L.I., Kim Y.A., Sukhorukov B.I. (2006). Influence of the temperature on polyelectrolyte microcapsules: Light scattering and confocal microscopy data. Russ. J. Phys. Chem. A.

